# Vitamin D receptor rs2228570 polymorphism is associated with LH levels in men exposed to anabolic androgenic steroids

**DOI:** 10.1186/s13104-018-3173-4

**Published:** 2018-01-19

**Authors:** Linda Björkhem-Bergman, Mikael Lehtihet, Anders Rane, Lena Ekström

**Affiliations:** 1 Division of Clinical Microbiology F68, Department of Laboratory Medicine, Karolinska University Hospital, Karolinska Institutet, Huddinge, 141 86 Stockholm, Sweden; 2 Department of Medicine, Division of Endocrinology, Metabolism and Diabetesm Karolinska Institutet, Karolinska University Hospital, Huddinge, 141 86 Stockholm, Sweden; 3 Division of Clinical Pharmacology, Department of Laboratory Medicine, Karolinska Institute, Karolinska University Hospital, Huddinge, 141 86 Stockholm, Sweden

**Keywords:** Vitamin D receptor, rs2228570, rs731236, Anabolic androgenic steroid, Hypogonadism, LH, FSH

## Abstract

**Objective:**

The primary aim of this study was to investigate the association between the vitamin D receptor polymorphisms rs2228570 (Fok1) and rs731236 (TaqI) and LH and FSH levels in relation to anabolic androgenic steroid (AAS) use.

**Results:**

Two cohorts were analyzed. Cohort 1 comprised healthy volunteers given single supra-physiological doses of 500 mg testosterone (n = 25). Cohort 2 comprised 45 self-reporting AAS users. Healthy volunteers homozygous for the C-allele of the Fok1 polymorphism exhibited 30% higher LH levels than T-carriers at baseline (p = 0.04) and twice the levels 14 days after testosterone administration (p = 0.01). AAS users homozygous for the C-allele had four times higher LH levels than TT-individuals (p < 0.05). FSH levels were not associated with Fok1 polymorphism, nor were LH and FSH levels associated with the TaqI polymorphism. In conclusion, there is an association between LH levels and the Fok1 VDR polymorphism and this difference is even more pronounced in AAS users and subjects with suppressed LH levels.

## Introduction

It is generally known that the use of anabolic androgenic steroids (AAS) suppresses the secretion of the pituitary luteinizing hormone (LH) and follicle stimulating hormone (FSH). This effect results from a negative feedback of androgens on the hypothalamic-pituitary–gonadal (HPG) axis. We recently showed that already a single dose of intra-muscular administration of testosterone or nandrolone suppresses the secretion of LH and FSH, but with large inter-individual variations [1, 2]. After discontinuation of AAS use, LH and FSH may be suppressed for a long period of time, resulting in anabolic steroid induced hypogonadism (ASIH) [3]. The time of AAS induced hypogonadotropic hypogonadism is highly variable and is dependent on the duration, dose and type of steroids used, co-use of other drugs and age, but may also be influenced by genetic factors [4–6].

Several studies have shown that vitamin D deficiency is associated with low testosterone levels in men [5, 7] which may be accompanied by low levels of gonadotropins [8]. Vitamin D exhibits its effect by binding to the vitamin D receptor (VDR). A previous study reported that a single nucleotide polymorphism (SNP) rs731236 (also known as TaqI) in VDR was associated with serum LH concentration but not with FSH concentrations in women with polycystic ovarian syndrome (PCOS) [9]. This polymorphism is a silent T > C SNP located in exon 9 and affects the function of VDR, probably due to an altered expression profile [10].

The VDR genotype rs2228570 (also known as Fok1*)* is a C > T non-synonymous polymorphism situated in the translation start site of VDR. The C variant results in a truncated protein, 3 amino acids shorter, with higher activity [11]. Studies have shown that carriers of the T variant, expressing a VDR-protein with lower activity, are more susceptible to different cancer forms, including ovarian cancer [12–15], and have an impaired immune system [16, 17]. However, this SNP has never been studied in relation to LH and FSH concentrations.

The aim of this pilot-study was to investigate the association between VDR rs2228570 and rs731236 polymorphisms and the levels of LH and FSH in relation to AAS use. To this end post hoc analysis of material from two previous performed studies were used.

## Main text

### Methods

Two cohorts of individuals were included; healthy volunteers given supra-physiological doses of testosterone (n = 25) and one group of self-reporting AAS users (n = 45). The study population of cohort 1 has been described earlier and included 25 male volunteers aged 27–43 years [2]. The participants were given a single intramuscular dose of 500 mg testosterone enanthate (Testoviron^®^ Depot) with the primary end-point to study different doses of testosterone and doping analysis in urine samples [18]. Exclusion criteria in the study were ongoing hormonal therapy treatment, treatment with NSAID, positive for hepatitis or HIV, being under the influence of abused substances, member of a Sports federation, malignancy within the last 5 years prior to study entry or allergy towards the compounds given. Blood samples were collected prior to (Day 0), and 4 and 14 days after testosterone administration. Blood samples were collected between 7.00 am and 9.00 am after an overnight fast. Cohort 2 consisted of AAS abusers between 18 and 57 years old. They were recruited to a previous study performed at our unit between 1993 and 2000 where men were asked to participate when they contacted the Anti-Doping Hot-Line, a free telephone counseling service for individuals abusing AAS [19]. A genuine desire to give up the abuse was a prerequisite to be included. Participation was commenced after informed consent, and no economical remuneration was given to the participants. A flow chart of the recruitment process has been published elsewhere [20]. Exclusion criteria in this study were not willing to stop their AAS abuse or not fluent in Swedish. Urine samples were collected and 84% of the participants were positive on AAS. The participants were clinically investigated and a series of endocrine parameters including LH and FSH were monitored in blood samples that were collected at the inclusion of the study.

LH and FSH were determined with accredited methods at the division of clinical pharmacology, at the time when the respective study was performed. In cohort 1 (analysed in 2014), FSH was measured by an immunochemical method on a Roche Cobas e602 after preparation with Elecsys FSH reagent kit (Cat no 11775863, Roche). LH was measured with AutoDELFIA using a AutoDELFIA hLH Spec kit (Cat No. 11732234, Perkin Elmer). Both methods had a lower detection limit of 0.05 IU/l. The samples from study 2 (analysed in 2000) were determined with electrochemiluminescence (Cobas 8000, Roche Diagnostics) with a lower detection limit 0.7 IU/l. The information of kits used at that time is not available.

Genomic DNA was isolated from 200 µl peripheral blood (Cohort 1) and from serum (Cohort 2) using the DNA Blood Mini kit (Cat No. 51104; Qiagen, Hilden Geramany). Allelic discrimination reactions were performed using TaqMan^®^ genotyping assays (Cat No. 4351379; Applied Biosystems, Foster City CA USA): C__12060045_20 for VDR (rs2228570); C___2404008_10 for VDR (rs731236). The final volume of each reaction was 10 µl consisting of 10–30 ng DNA and 2×Taqman Universal PCR Master mix (LifeTechnology, Applied Biosystems, Catalog no 4440047). The PCR profile consisted of 95 °C for 10 min followed by 40 cycles of 92 °C for 15 s and 60 °C for 1 min. The fluorescence signal was measured with an ABI 7500 Sequence detector (Applied Biosystems).

All statistical tests were performed using GraphPad Prism (San Diego, California, USA) v. 6.00 and values of p ≤ 0.05 were considered statistically significant. Since the hormone levels of gonadotropins are not normally distributed, non-parametric Mann–Whitney U test was used.

### Results

**Fig. 1:**
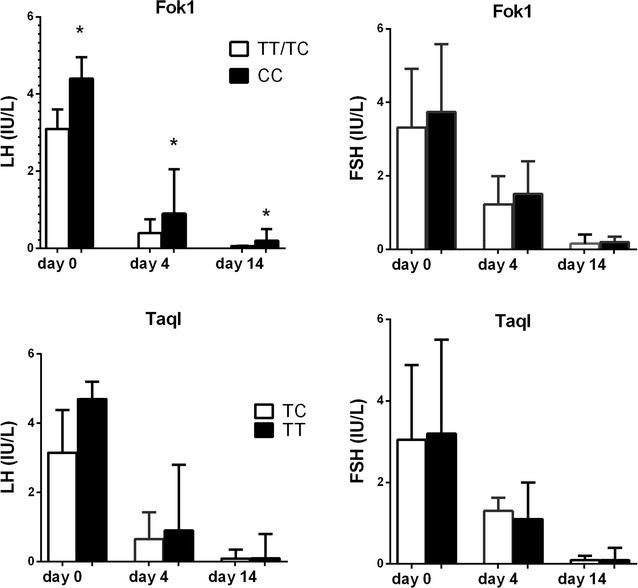
The suppression of **a** LH and **b** FSH 4 and 14 days after the administration of testosterone (cohort 1, n = 19) in relation to VDR Fok1 (rs222857 C < T) and TaqI (rs731236 T < C) SNPs. Column shows mean values and bars SD. * p < 0.05

In Cohort 1 the genotype frequencies were Fok1 CC = 53%, TC = 26% and TT = 21% and for TaqI TT = 37%, TC = 63% and CC = 0%, respectively. The Fok1 polymorphism was associated with the LH concentration at baseline as well as after 4 and 14 days after the testosterone dose. Individuals homozygous for CC had 30% higher LH concentration at baseline compared to T-carriers; mean 4.2 ± 0.9 compared to 3.2 ± 1.2 (p = 0.04). At 4 and 14 days, the LH concentrations were approximately twice as high in the CC subjects compared to the T-carriers; mean 1.5 ± 1.3 and 0.33 ± 0.29 compared to 0.73 ± 0.71 and 0.18 ± 0.10, (p = 0.04 and p = 0.01) (Fig. 1). There was no association between the Fok1 SNP and FSH concentration at any time (Fig. 1). The TaqI VDR genotype (rs731236) was not associated with LH and FSH concentrations at any of the time points (Fig. 1). The genotype frequencies of both SNPs were in Hardy–Weinberg (HW) equilibrium.

In order to confirm the findings in Cohort 1 we genotyped samples obtained from AAS users. When the subjects showed up for the first visit, serum samples were collected. The time span from their last intake of AAS varied between a few days and some months. The genotype frequencies of TaqI were TT = 49%, TC = 42% and CC = 9% and for Fok1 see Table 1. In summary, the LH levels were significantly lower than the baseline levels observed in the healthy volunteers (p < 0.001) as a result of their AAS use. A significant association between the Fok1 VDR polymorphism and the LH concentration was found also in Cohort 2, whereas no association was found between this SNP and the FSH concentration (Table 1). Individuals homozygous for the C-allele had significantly higher LH levels than carriers of the T-allele (p < 0.05) and four times higher levels than TT-individuals (p < 0.05). The genotype frequency of rs2228570 was in HW equilibrium, whereas the genotype frequency of rs731236 was not (p = 0.04).

**Table 1:** LH and FSH concentrations (mean ± SD) in self-reporting AAS users from cohort 2 (n = 45) in relation to VDR rs2228570 polymorphism

VDR Genotype	LH (IU/l)	FSH (IU/l)
rs2228570		
TT (n = 7)	0.60 (± 0.60)	0.35 (± 2.24)
TC (n = 21)	1.68 (± 1.35)	1.40 (± 1.82)
CC (n = 17)	2.20 (± 0.59)*	1.20 (± 1.34)

CC-individuals had statistical significant higher LH-levels compared to individuals carrying the T-allele

* p < 0.05

### Discussion

The results from this study show that individuals homozygous for the T-allele of the VDR polymorphism Fok1 (rs2228570) have lower circulatory LH levels than carriers of the C-allele in two different populations. The difference in LH concentration between the genotype groups was larger after the administration of a supra-physiological dose of testosterone and in self-reporting AAS users as compared to baseline values. This is the first time a VDR polymorphism is shown to be associated with serum levels of LH in relation to AAS use.

The Fok1 (rs558750) C-allele leads to a truncated protein, three amino acids shorter than the wildtype T-variant. The C-allele has been associated with higher activity in vitro [11]. Subjects with the T-allele, i.e. expressing the protein with lower activity, have been shown to have increased risk of hormonal dependent cancers, such as ovarian [14, 21] and prostate cancer [22]. The association with breast cancer seems to be weaker and different studies have shown divergent results [23–25].

It has previously been reported that serum LH concentrations in women with PCOS are associated with the VDR TaqI (rs731236) polymorphism [9]. PCOS is a disorder where women have naturally higher concentrations of circulatory androgens as well as LH [26, 27]. We could not find any association between the rs731236 polymorphism and gonadotropin concentrations in the cohorts studied here. It is possible that the VDR genotypes exert different gender and/or disorder effects [28]. Based on these data it seems that androgens, whether endogenous or exogenous seem to amplify the genetic association with the circulatory LH concentrations. A recent GWAS study including 2913 individuals (294 males) identified that a genetic variant near the FSHB gene was associated with LH levels [29].

LH is produced exclusively in the gonadotropic cells of the anterior pituitary. VDR is abundant in the human pituitary [30] and it is plausible that VDR may be involved in the synthesis of gonadotropins and that the more efficient VDR C-allele is associated with an increased transcriptional activity of genes required for the synthesis of LH subunits. FSH levels were not affected by the VDR polymorphism. FSH is partly regulated by other mechanisms than LH. For example, inhibin and activin from the sertoli cells affects the FSH levels independently of androgens.

Several studies have shown that vitamin D deficiency is associated with low testosterone levels in men [5, 7], which may be accompanied by low levels of gonadotropins [8]. Wehr et al. hypothesized that vitamin D may have an impact directly on the gonadal function since testosterone and vitamin D displayed a concordant seasonal pattern [31]. Moreover supplementation with vitamin D increases the total testosterone levels in some studies [32]. Therefore, it is of clinical interest to further investigate how vitamin D and the VDR mediated effect is involved in the regulation of the HPG-axis.

Supra-physiological doses of androgens are known to suppress LH and FSH with a fast response that may persist for a long time [3]. The resulting hypogonadism has been associated with the more distressing AAS induced side effects, such as depression and sexual dysfunction [33, 34]. The degree of suppression and the ability to recover after an AAS cycle depends on the duration of AAS use and the cumulative dose [35].

However, these findings are not only of interest in relation to AAS abuse but also to men with iatrogenicaly suppressed LH-levels, as in prostate cancer patients on hormonal therapy. Indeed, the Fok1 VDR polymorphism has been associated with the prognosis in prostate cancer [36]. If the results presented here also apply to women it may indicate that subjects homozygous for the T-allele would have an even higher suppression of their LH-levels when taking contraceptives than in subjects with the C-allele. The results might also contribute to the understanding of the increased risk of ovarian cancers in women with the T-allele [14, 21].

In conclusion, we show an association between LH levels and the Fok1 VDR polymorphism and that this difference is even more pronounced in AAS users and subjects with suppressed LH levels. The role of VDR in the synthesis and secretion of LH warrants further studies.

## Limitations

This study has some limitations that need to be considered. The population included male subjects who self-reported AAS use, different doses and administration routes have been used, and the time elapsed since their last AAS intake varied between some days to some months. However, it is not possible to conduct a controlled study using the high doses AAS users normally use. In human studies, lower doses and/or single doses are given. Another limitation is the small number of subjects included. Still, the same findings are present in both cohorts although they are collected with more than 10 years in between. The hormone analysis in Cohort 2 was analyzed in year 2000 and the method used at this time was not as sensitive as the methods used today. The lower detection limit for LH was then only 0.7 IU/l. It is possible that with a more sensitive method employed today an even larger association of the Fok1 genotype and LH secretion could have been discerned.

## Abbreviations

AAS: anabolic androgenic steroids
ASIH: anabolic steroid induced hypogonadism
FSH: follicle stimulating hormone
LH: luteinizing hormone
SNP: single nucleotide polymorphism
VDR: vitamin D receptor
